# Graphene Flake Self-Assembly Enhancement via Stretchable Platforms and External Mechanical Stimuli

**DOI:** 10.1021/acsomega.1c04368

**Published:** 2021-11-05

**Authors:** Harrison A. Loh, Claudio Marchi, Luca Magagnin, Konstantinos A. Sierros

**Affiliations:** † Statler College of Engineering and Mineral Resources, West Virginia University, Morgantown, West Virginia 26506, United States; ‡ Department of Chemistry, Materials and Chemical Engineering Giulio Natta, Politecnico di Milano, Via Mancinelli 7, 20131 Milano, Italy

## Abstract

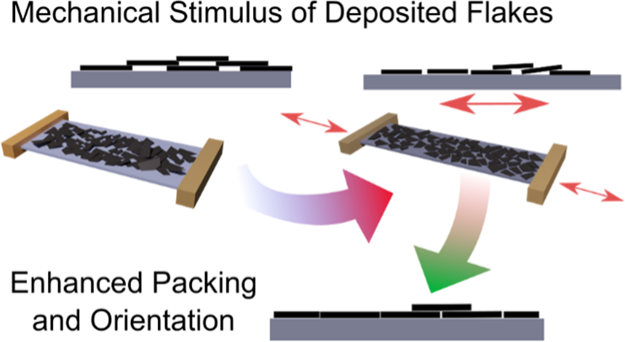

While the green production and application of 2D functional nanomaterials, such as graphene flakes, in films for stretchable and wearable technologies is a promising platform for advanced technologies, there are still challenges involved in the processing of the deposited material to improve properties such as electrical conductivity. In applications such as wearable biomedical and flexible energy devices, the widely used flexible and stretchable substrate materials are incompatible with high-temperature processing traditionally employed to improve the electrical properties, which necessitates alternative manufacturing approaches and new steps for enhancing the film functionality. We hypothesize that a mechanical stimulus, in the form of substrate straining, may provide such a low-energy approach for modifying deposited film properties through increased flake packing and reorientation. To this end, graphene flakes were exfoliated using an unexplored combination of ethanol and cellulose acetate butyrate for morphological and percolative electrical characterization prior to application on polydimethylsiloxane (PDMS) substrates as a flexible and stretchable electrically conductive platform. The deposited percolative free-standing films on PDMS were characterized via in situ resistance strain monitoring and surface morphology measurements over numerous strain cycles, with parameters extracted describing the dynamic modulation of the film’s electrical properties. A reduction in the film resistance and strain gauge factor was found to correlate with the surface roughness and densification of a sample’s (sub)surface and the applied strain. High surface roughness samples exhibited enhanced reduction in resistance as well as increased sensitivity to strain compared to samples with low surface roughness, corresponding to surface smoothing, which is related to the dynamic settling of graphene flakes on the substrate surface. This procedure of incorporating strain as a mechanical stimulus may find application as a manufacturing tool/step for the routine fabrication of stretchable and wearable devices, as a low energy and compatible approach, for enhancing the properties of such devices for either high sensitivity or low sensitivity of electrical resistance to substrate strain.

## Introduction

Green and flexible electronic technologies are attracting an ever-increasing amount of attention as an alternative for current rigid technologies due to their potential for realizing unique form factors, multifunctionality, improved sustainability, and greater conformability for incorporation into applications such as optoelectronics, soft robotics, and biomonitoring.^1−3^ The utilization of graphene as an active component in these next-generation technologies provides an additional improvement, given the high theoretical electrical and thermal conductivities of this material.^4^ The liquid-phase exfoliation (LPE) method for graphene flake production has been investigated as a promising method, which seeks to address the need for a method that is scalable and environmentally friendly.^5^ LPE provides an alternative or supplement to approaches such as mechanical exfoliation of flakes (i.e., using some form of adhesive to peel away graphene layers),^6^ chemical vapor deposition (CVD),^7,8^ and employing graphene-oxide (GO)/reduced GO materials.^9,10^ LPE has been reported using a variety of solvent systems, particularly polymer solutions such as ethylcellulose and ethanol (EtOH), polyvinylpyrrolidone and water, and nitrocellulose and acetone.^11−13^

One continuous challenge for graphene flake technologies with stretchable substrates is processing methods for enhancing the electrical conductivity of these percolative flake films. For traditional, rigid substrates, a high-temperature annealing step is often required to reduce the presence of insulating elements such as residual adsorbed solvents and polymers/other non-functional components.^14,15^ In contrast, flexible substrates and stretchable platforms are not readily compatible with such thermal processing, as the structural stability of these substrate materials is compromised due to decomposition and increased stiffening at relatively high temperatures. This thermal limitation requires the application of high but concentrated energy alternatives such as focused laser annealing and rapid photonic annealing to reduce the electrical resistivity.^11,16,17^ Therefore, alternative low-energy processing approaches are crucial for enhancing the conductive transport in graphene films on flexible substrates.

A common substrate for stretchable and wearable devices is polydimethylsiloxane (PDMS). PDMS has often been reported for the fabrication of devices pertaining to a diverse range of applications, both with graphene flakes and other morphologies of nanomaterials. Examples include electro-jetting deposition on PDMS substrates,^18^ a flexible platform for NO_2_ gas sensors,^19^ a wearable strain sensor with silver nanoparticles,^20^ and printable graphene planar networks as stretchable strain detectors.^21^ Conventionally, most experiments exploring the application of strain with PDMS consist of measuring the maximum strain that flexible devices can handle until failure. Conversely, lower ranges of cyclically applied strain may serve to reshuffle or reorganize the overlaying flake material, given the tribological differences between graphene flakes on PDMS versus graphene flakes stacked on top of graphene.^22^ Therefore, we hypothesize that applying a mechanical stimulus to the stretchable platform can increase the packing density and optimize the orientation of the deposited flakes through strain transfer from the substrate to the adjacent loosely adhered material. In this way, the applied mechanical strain can be employed as a potential tool for modulation of the properties of such films.

In this study, we focus on the following: first, exfoliation of graphene flakes in EtOH with the assistance of cellulose acetate butyrate (CAB) is demonstrated as a green approach. These flakes are then characterized with respect to the morphology and percolative electrical characteristics on SiO_2_ substrates, which serves as a foundation for understanding their properties when applied to flexible conductive films. Second, strain-induced restacking and optimization of these graphene flakes on stretchable substrates is investigated. PDMS was chosen as a model stretchable platform to study strain-induced modification. The use of mechanical strain as a low-energy processing approach is demonstrated to enhance the conduction of graphene flake films on PDMS substrates. The corresponding modification of properties such as the surface morphology and resistive-strain behavior of strained samples contributes to the hypothesis that surface reorganization of the graphene flakes is the primary mechanism for electrical behavior changes.

## Results and Discussion

### Exfoliation of Graphene in CAB/EtOH

The exfoliation of graphene flakes in a polymer/alcohol mixture has been reported as a successful strategy to obtain graphene flakes.^11−13^ As a biocompatible and biodegradable polymer, CAB has been used in pharmaceutical and biomedical applications,^23^ in gas-sensing layers,^24^ and as an additive in graphene inks.^25^ CAB has been shown to leave a reduced residue on graphene films when compared to poly(methyl methacrylate) when used as both a masking material and a transfer polymer for CVD-grown films.^26^ This reduction in the polymeric residue combined with the biofriendly nature of CAB would enable its utilization as a steric stabilizer for green exfoliation, which currently has not been demonstrated.

**Figure 1:**
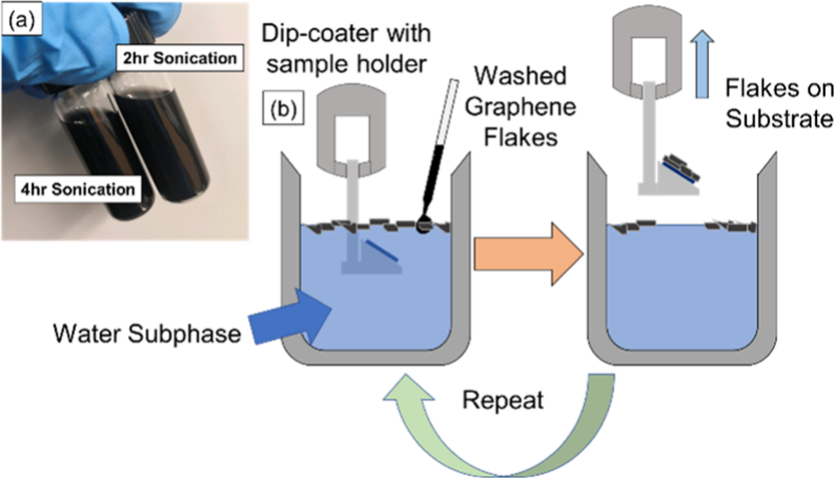
(a) Stably dispersed graphene flakes in EtOH and CAB for two different sonication intervals. (b) General procedure for surface self-assembly and deposition of washed graphene flakes onto substrates. The utilization of a commercial dip-coating machine allows coating of different types of substrates by changing the form of the sample holder.

In the present work, CAB/EtOH provided very stable dispersions, which showed no visible sedimentation for over 4 months (Figure 1a). The removal of excess CAB through centrifuging and substitution with fresh EtOH is necessary to ensure optimal self-assembly of flakes, and despite the reduction in the shelf-life of the dispersions, a brief sonication of the mixture for ∼1–2 min is sufficient to redisperse. Thermogravimetric analysis (TGA) was employed to quantify the polymeric residue, which was estimated at ∼5 wt % for the final 2 h exfoliated material and at ∼10 wt % for the 4 h LPE process. The TGA curve of CAB shows significant initial decomposition at ∼380 °C, with a second peak closer to 526 °C. From this, 500 °C was deemed the optimal temperature to ensure CAB removal in samples deposited on SiO_2_/Si (Supporting Information Figure S2).

The efficiency of the exfoliation process was estimated by vacuum filtering and weighing of the obtained material. The 2 h sonication route provides ∼1.02 mg of graphene (68 μg mL^–1^), with an efficiency of 0.133%; conversely, the 4 h process provides ∼2.8 mg of graphene (186 μg mL^–1^) with an efficiency of 0.35%. Mass yields were based on a starting graphite amount of 0.8 g. While these yields and efficiencies are somewhat lower than what have been reported in other works employing liquid exfoliation approaches,^33,34^ further processing and recycling of the unexfoliated graphite material can provide an additional increase to the total efficiency and yield.

### Raman Spectroscopy of Exfoliated Graphene

Raman spectroscopy of LPE graphene has been reported as a common metric for graphene, as it is generally a quick, non-destructive metrology tool for characterization of structural and electrical characteristics. Raman spectra of graphene and graphene-like materials commonly consist of three primary features/peaks. These are (from smaller to higher wavenumbers) the D peak, the G peak, and the 2D/G′ peak. The D peak is commonly referred to as the “defect peak” because its presence suggests a break in the symmetric ordering of the graphene structures (related to the Raman selection rules), either suggesting basal plane defects (e.g., vacancies) or edge-type defects (e.g., grain boundaries and flake edges).^35,36^ The G peak is present in many types of carbon materials and results from the first-order Raman scattering at the Brillouin zone center due to the doubly degenerate iTO and LO phonon modes.^37^ The 2D/G′ peak is due to a second-order Raman process originating from in-plane breathing modes of the aromatic structures of graphene.^38^ For additional explanations of the origins of these Raman peaks as well as phonon dispersions in graphene in general, further reviews are recommended to the interested reader.^37−40^

**Figure 2:**
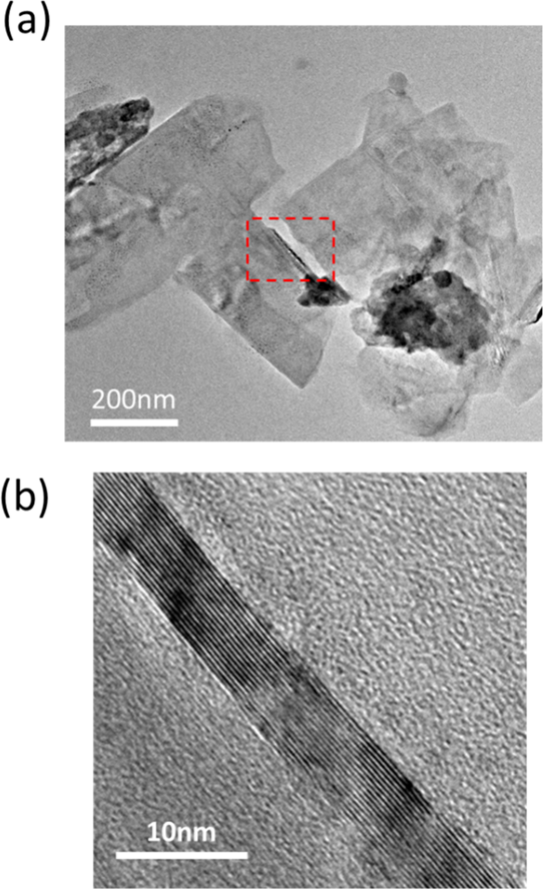
TEM images of a multilayer graphene flake after 4 h of sonication in CAB/EtOH. The multilayer structure at the edge [boxed portion of (a)] of the flake can be seen in the close-up in portion (b).

**Figure 3:**
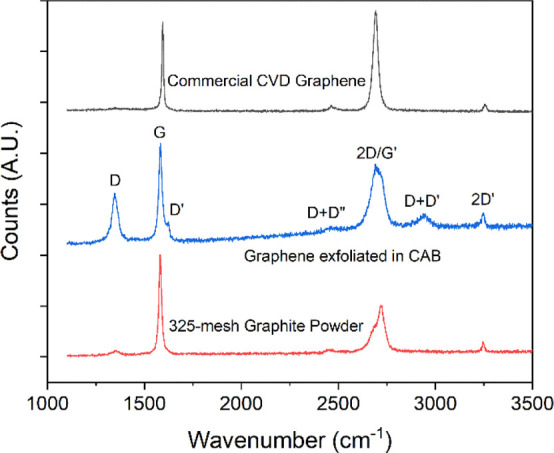
Representative Raman spectrum of graphene exfoliated in CAB for 2 h, along with the spectra of commercial CVD graphene and the precursor graphite powder for comparison. All spectra are scaled to the intensity of the G peak.

In LPE graphene, the lateral flake dimensions are generally on the order of (or smaller than) the spot size of the laser (∼3 μm diameter for the power used), resulting in flake edges commonly being probed for any given sample measurement and thus resulting in a significant D peak a low presence of basal plane defects for any given exfoliated flake. For example, the flakes produced by 4 h of sonication gave an average length of 810 nm ± 358 nm along their long axis (Figure 2). Graphene flakes exfoliated in CAB have similar features as well as additional combinations or overtones/harmonic features when compared to the precursor graphite powder and CVD-grown graphene films (Figure 3). Both graphite, commercial CVD graphene, and the CAB-exfoliated graphene flakes possess strong G and 2D peaks, as expected with graphite/graphene materials, with the addition of peaks at ∼2450 cm^–1^ corresponding to the combination mode D + D″ peak and the overtone 2D′ peak at ∼3240–3248 cm^–1^. The D′ shoulder arising near the G peak and the D + D′ combination mode peak are unique features of the Raman spectra of the exfoliated graphene as they are absent when compared to the other two spectra.

The occurrence of the D + D′ peak in the exfoliated samples compared with the other two types of Raman spectra in Figure 3 can be a result of the enhanced contribution from the D peak, which is virtually absent in CVD graphene (due to large grain sizes and the presence of few boundary defects) and only mildly present in the precursor graphite powder [due to large initial flake sizes and comparatively larger spot size than the Raman laser spot size (Supporting Information Figure S3)]. This D peak combined with the D′ shoulder results in the D + D′ combination mode peak.

A final spectral difference to note between the exfoliated graphene and graphite/CVD graphene spectra is the line shape of the 2D/G′ peak, which has been utilized in a number of different quantitative analyses for graphene, such as estimating the layer numbers.^41^ While the 2D line shape and fitting are fairly well understood for the low number of graphene layers (i.e., 1–3L; where L = layer), the line shape and fitting practices become more convoluted and diverse with increasing layer numbers, as well as when attempting to fit and analyze the Raman spectra obtained from ensembles of sheet-like materials of varying sizes and thicknesses.^42^

The line shape of the 2D peak presented in Figure 3 may be interpreted as corresponding to graphene of ∼3–5L, based on published 2D line shapes for the Raman spectra collected at 532 nm excitation.^41^ The primary characteristic that corresponds to these layer numbers is a relatively symmetrical line shape, with a slight tilt at the 2D peak toward the lower wavenumbers. Spectra collected for graphene films via self-assembly and modified dip-coating deposition possess a line shape qualitatively closer to the ∼5–7L graphene line shape (Supporting Information Figure S4). Thicker flakes on the order of 15–20L are also present though, as seen from the transmission electron microscopy (TEM) image in Figure 2, the frequency of which may be modified through incorporation of tighter centrifugation process parameters or other size-filtering methods.

### Modified Langmuir–Blodgett Assembly Method

The coating method employed for graphene percolative films is a modified version of a traditional dip-coating procedure based on the Langmuir–Schaefer (LS) approach (Figure 1b). Recently, a modification of the LS approach (modified LS, m-LS) has been reported for coating hydrophilic as well as hydrophobic substrates by submerging the substrate of interest below the subphase, assembly of the exfoliated material on the subphase surface, then draining the liquid subphase, and effectively lowering the assembled materials to “drape” over the substrate.^27−31^ The modified self-assembly deposition method relies upon the Marangoni effect taking place between two liquids: the water subphase and EtOH acting as the dispersing solvent for the graphene flake dispersion.^32^ The Marangoni effect can be described as a mass transfer at the interface between two liquid phases induced by a surface tension gradient. For simple cases, the speed of the interfacial flow can be approximated as *u* = Δγ/μ, with γ being the surface tension and μ being the viscosity of the subphase. The choice of water as the subphase was made aiming at the maximization of the Δγ value, roughly 50 mN/m for the EtOH/water system. The thorough washing of the graphene flakes was needed to minimize the polymeric agglomerates generated by the interaction of CAB with water.

### Raman Spectroscopy of Deposited Films

**Figure 4:**
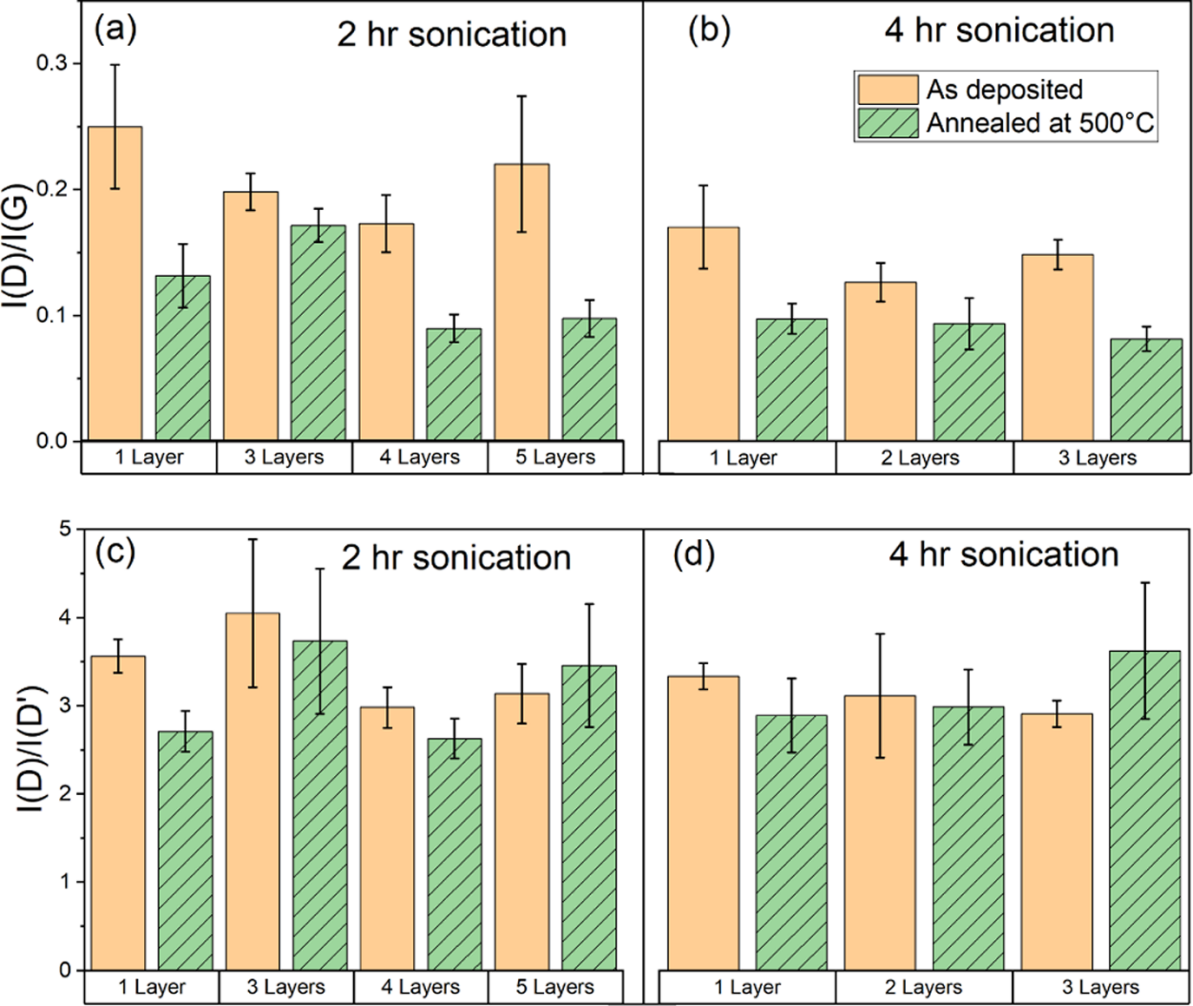
Evolution of the intensity ratios of (a,b) *I*(D)/*I*(G) and (c,d) *I*(D)/*I*(D′) for the self-assembled film’s Raman spectra as a function of layers deposited with/without thermal treatment. Sample designations are made based on the hours of sonication, followed by the number of deposition iterations or “layers”.

Two numerical quantities were chosen to quantify the layer and thermal-based changes in the deposited graphene flake films: the ratio of the D band intensity to the G band intensity [*I*(D)/*I*(G)] and the ratio of the D band intensity to the D′ peak intensity [*I*(D)/*I*(D′)] (Figure 4). A Fano line shape was used to fit the D′ peak, while Lorentzian functions were used to fit the D and G peaks.^36^ While the D band did not vary significantly in line shape between samples, the G peak occasionally exhibited slight splitting, the origin of which is still under investigation. In these cases, the composite intensity (i.e., maximum intensity of the entire peak) was used to calculate the ratios described above.

While the I(D)/I(G) ratio has been employed as a “quality” metric for graphene, it is dependent on the excitation energy.^43^ Despite the fact that this introduces difficulty in potential comparisons between works with different Raman laser wavelengths, it allows for comparison between the samples presented in this work as the same spectrometer power and excitation laser wavelength were used for all samples.

For both 2 and 4 h sonicated film samples, thermal treatment at 500 °C results in a definitive decrease in the D/G peak ratio (Figure 4a,b). Though the decrease was not uniform for each sample, the main contribution is expected to be a result of thermal removal of adsorbed functional groups and restoration of sp^2^ domain areas.^44^ In spite of the fact that a reduction in the D/G ratio can be attributed to a reduction of flake edges, which have been shown as a large contributor to the D-peak during tip-enhanced Raman measurements across graphene flakes,^45^ such a contribution is expected to be negligible for far-field Raman measurements.

The *I*(D)/*I*(D′) ratio was used as a metric for defect types present in the exfoliated graphene flakes. This metric has been reported for distinguishing between the sp^3^-type (*I*(D)/*I*(D′) ∼ 13), vacancy-type (*I*(D)/*I*(D′) ∼ 7), and boundary-type (*I*(D)/*I*(D′) ∼ 3.5) defects in graphene materials.^36^*I*(D)/*I*(D′) measurements for samples were found to center around 3.5, suggesting a vast majority of boundary-type defects in the prepared samples, which correspond to the flake edges in the exfoliated graphene (Figure 4c,d). Thermal treatment was found to have a minimal effect on this ratio, particularly the lack of a significant increase in the ratio with thermal treatment suggests the absence of vacancy- and sp^3^-type defect generation with heating.

### X-ray Photoelectron Spectroscopy of Deposited Graphene Flakes

To confirm the absence of significant sp^3^-type defect generation as well as the presence of any other oxidation, X-ray photoelectron spectra were collected from two samples (one 2 h and one 4 h sonicated) as representative measurements for the two different sonication times. Charging effects were compensated for by shifting the binding energy of the dominant C 1s peak to 284.8 eV to correspond to sp^2^ C–C bonding. Survey spectra of film samples showed expected peaks related to carbon (C 1s), oxygen (O 1s, O KLL), as well as silicon from the underlying SiO_2_/Si substrate (Si 2s, Si 2p) (Supporting Information Figure S5). C 1s high-resolution measurements of the samples (Supporting Information Figure S6a,b) show additional peaks in the 2 h sonicated sample compared to the 4 h sonicated sample, which have been assigned to carboxyl groups (C=O(OH)), hydroxyl/epoxide groups (C–OH and C–O–C), and sp^3^ C–C bonds in the graphene basal plane.^46−48^ Similar peaks were not observed in either the as-deposited 4 h material or the annealed 4 h material. A comparison between the *I*(D)/*I*(G) ratios of both samples used for XPS also shows a lower ratio for the 4 h sonicated sample compared to the 2 h sonicated material. Subsequent calculations of O/C ratios for the as-deposited samples and samples after thermal treatment were done after subtracting oxygen contributions from the underlying SiO_2_ substrate (Supporting Information Figure S6c). As confirmation to the abovementioned correlation between the oxidation group peaks and the Raman data for 2 h sonication, the O/C ratio was also found to be lower for the 4 h sonicated graphene flakes compared to flakes that were sonicated for 2 h.

### Electrical Characterization of Percolative Films

Four-point sheet resistance measurements were performed for both the 2 and 4 h LPE processes on SiO_2_/Si substrates, starting from the first conductive layer and for two successive deposition iterations. The exfoliated graphene flakes obtained after a 2 h exfoliation process required three layers to provide a stable resistance measurement, hence the set of samples was made of 3L, 4L, and 5L films (referred to as 2 h 3L, 2 h 4L, and 2 h 5L, respectively). Conversely, the 4 h exfoliated graphene dispersion produced a set of samples consisting of 1L, 2L, and 3L films (denoted 4 h 1L, 4 h 2L, and 4 h 3L, respectively). Sheet resistance values for both sets of samples before and after annealing on SiO_2_ wafers are shown in Supporting Information Figure S7.

**Figure 5:**
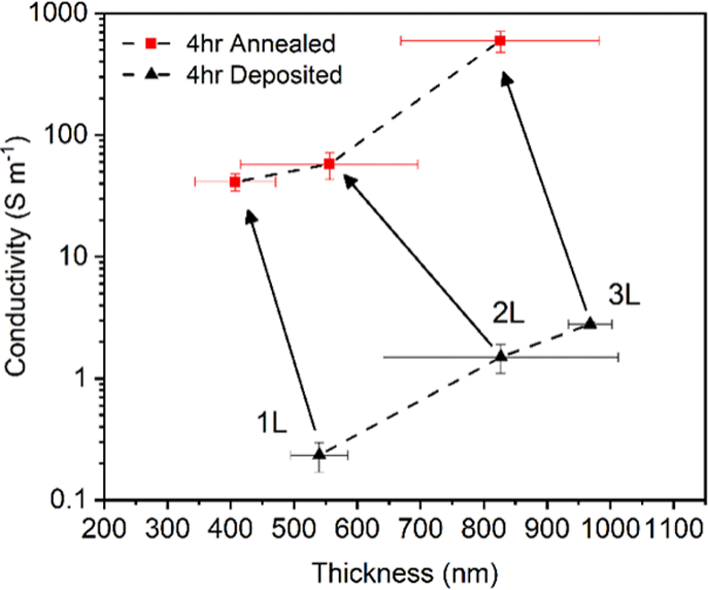
Conductivity of percolative films of graphene flakes (4 h sonication) on SiO_2_/Si substrates. The multiple iterations of dip-coating from 1L up to 3L show corresponding increases in both the film thickness and conductivity. A significant increase of 2 orders of magnitude in conductivity as well as a reduction in film thickness are seen when heating the samples to 500 °C.

Thermal annealing performed at 500 °C for 60 min removed the CAB polymeric residues and the remaining solvent, thus resulting in a significant reduction of the observed sheet resistance, in the range from MΩ cm^–1^ to kΩ cm^–1^, which are in line with values reported for film-fabrication methods based on exfoliated graphene such as Langmuir–Blodgett (LB), vacuum filtering, and inkjet printing.^49−53^ Additional characterization of the 4 h sonicated graphene flakes (which had a greater reduction in sheet resistance with layer thickness compared to the 2 h sonicated flakes) shows an increase in the conductivity with the thicker films for both the as-deposited and annealed samples (Figure 5) along with a corresponding decrease in the film thickness when heated.

### Surface Morphology and Electrical Changes with Applied Strain

**Figure 6:**
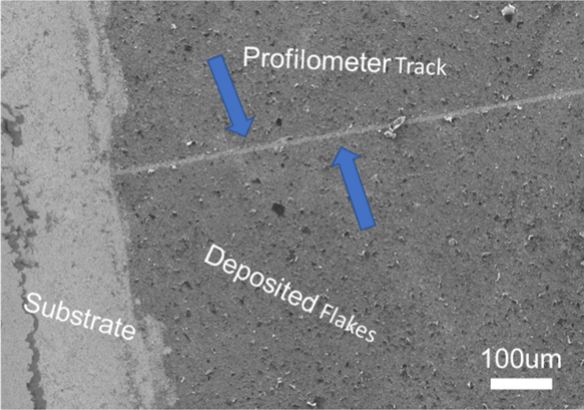
SEM image of the surface of the 4 h 2L sample following examination by stylus profilometry. The track made by the stylus across the surface, emphasized by the blue arrows, highlights the loose adhesion of the dip-coated material to the underlying substrate.

As the 4 h sonicated material showed a significantly stronger reduction in resistance with thicker films (with a maximum conductivity of ∼600 S m^–1^ achieved for the annealed 3L sample) compared to the 2 h sonicated sample, samples for investigating the hypothesis that substrate straining resulting in flake reorganization were prepared using graphene sonicated for 4 h in CAB/EtOH. The consistent reduction of the film thickness and increase in the conductivity make the 4 h sonicated material a model material to study the potential of applied strain to enhance the transport of films on flexible and stretchable substrates. For dip-coated films on both SiO_2_/Si and PDMS, the mechanism for substrate adhesion can be assumed to be primarily due to van der Waal (vdW) forces, with the adhesion of subsequent layers on top of the prior-deposited layers also due to vdW forces between the basal plane and edge atoms in the exfoliated flakes, depending on the orientation during deposition. To confirm this, substrate adhesion was briefly investigated utilizing stylus profilometry (Dektak, 2 μm radius stylus, 1 mg force) as an unconventional scratch test for a 4 h 2L sample on SiO_2_/Si (Figure 6). The apparent wear track in the sample film confirms the weak substrate interaction present due to the dip-coating process employed and that an external stimulus such as mechanical force may induce mobility in the exfoliated flakes.

**Figure 7:**
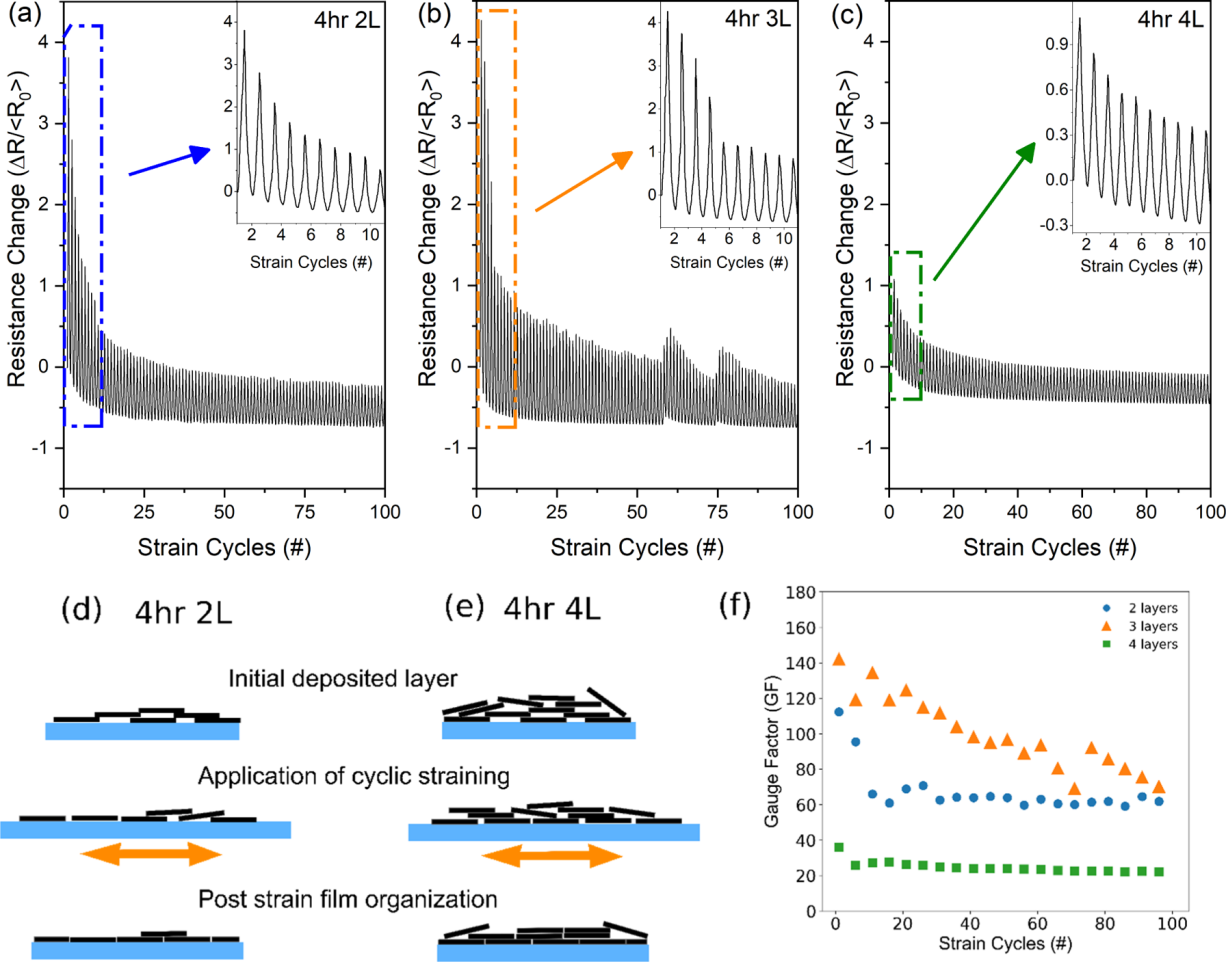
(a–c) Normalized resistance change of 4 h 2L, 3L, and 4L on PDMS substrates. A strain of 3% was applied to the substrate over 100 cycles, during which a decrease in the magnitude of resistance change, as well as the unstrained resistance, was observed for all samples. Inset plots in (a–c) focus on the first 10 cycles of strain. (d,e) Proposed mechanism for the resistance drop seen over the course of the cyclic straining of the PDMS samples. The straining of the substrate (blue) results in shear stress transfer to the overlaying flakes, forcing the platelet material to slide and move around each other before reaching an equilibrium state. (f) GF calculation for each sample over the course of straining. A reduction in the GF is seen for all samples, with the smallest reduction occurring in the 4L sample.

**Figure 8:**
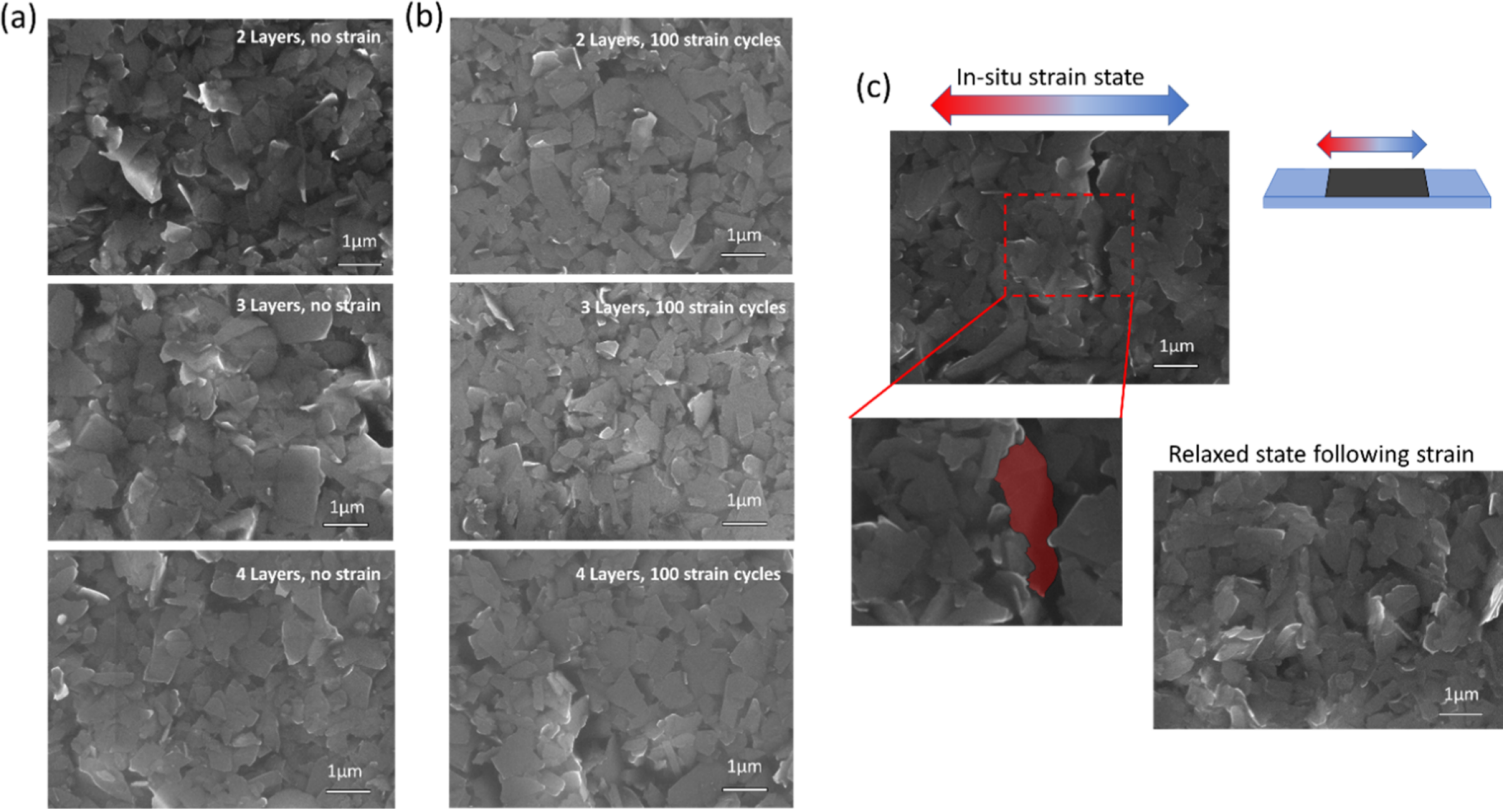
SEM imaging of exfoliated flakes under different strain processing states to observe the changes in the surface morphology. (a) Initial deposition showing a higher degree of out-of-plane oriented flakes as well as some examples of surface void spaces between particles. (b) Following the applied strain, the surface packing increases with a lower occurrence of out-of-plane alignment. (c) In situ SEM images collected during initial straining show spacing between areas of the sample, allowing particles (false colored red) to migrate into these open spaces prior to sample relaxation.

**Figure 9:**
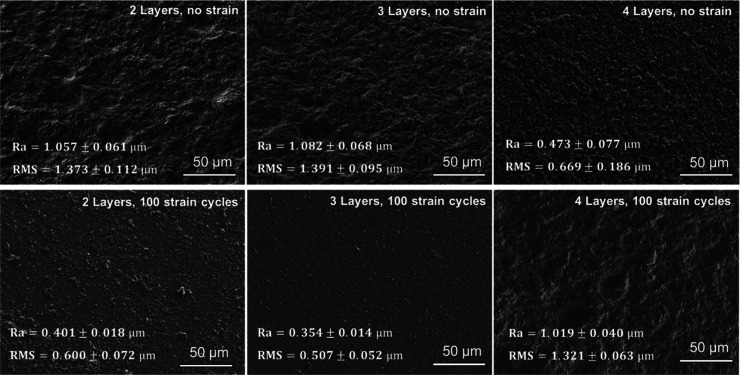
Lower-magnification SEM images of exfoliated graphene flakes on PDMS substrates. Significant morphology changes to the surface roughness of the samples can be seen when comparing the as-deposited samples (no strain) to those samples subjected to 100 cycles of 3% tensile strain. A reduction in the root arithmetic (Ra) and root mean square (rms) roughness is seen for the 2L and 3L samples, whereas the 4L sample shows an increase in roughness compared to its non-strained counterpart.

For illustrating this concept of mechanical stimuli for surface reorganization, 4 h exfoliated flakes were deposited on PDMS substrates through the same dip-coating process as for SiO_2_ and subjected to 100 cycles of tensile straining at 3% strain while logging the two-point resistance (Figure 7). The maximum strain of 3% was chosen based on comparable strain limits in other reports for graphene films for flexible electronics^54,55^ as well as the strain limit for ceramic brittle films.^56^ Scanning electron microscopy (SEM) (Figure 8) and surface roughness measurements from optical profilometry (Figure 9, Supporting Information Figure S8) were collected from the as-deposited samples and samples following strain processing. For the samples made, the electrical resistance of the 1L sample was found to be outside the measurement range of the digital multimeter used, and, as such, samples beginning with two dip-coated layers and ending at four dip-coated layers were tested to determine the effect of film thickness on the nature of resistance reduction and surface film reorganization. Inset plots in Figure 7 provide a closer view to emphasize the profile of the resistance change for the first 10 cycles performed, with Table 1 providing additional parameters extracted from the resistance–strain curves. For reference, the average initial resistances (⟨*R*_0_⟩) of the samples on PDMS from the 15 s startup measurements were 7.69 ± 0.03, 5.19 ± 0.01 MΩ, and 267.97 ± 0.29 kΩ for the 2L, 3L, and 4L samples, respectively.

**Table 1:** Resistance Cycle Characteristics for Samples of Different Thicknesses^a^

sample	minimum ΔR/⟨R0⟩	minimum difference between strained and relaxed ΔR/⟨R0⟩	cycle where 90% of minimum Δ*R*/⟨*R*0⟩ is reached
2L	–0.726	0.493	26
3L	–0.745	0.537	23
4L	–0.453	0.362	42

a The more negative the minimum Δ*R*/⟨*R*_0_⟩ value, the greater the resistance drop between the first and last strain cycles.

From observing the normalized resistance change, a significant change is seen over the course of the strain cycles measured (Figure 7a–c). For the 2L and 3L samples, the first strain cycle shows a resistance change up to ∼4 times the initial resistance, with a decrease in both the change in the resistance between unstrained and strained states [i.e., gauge factor (GF)] as well as a shift in the resistance value of the sample at ϵ = 0 (i.e., no strain). For the 4L sample, both the overall resistance and the rate at which the resistance changed are significantly smaller than those of the other two samples measured.

The gauge factor of the samples was calculated as a function of the strain cycles applied by considering the initial and peak resistance for each cycle (Figure 7f). For the samples tested, the 3L sample exhibited the largest initial GF of ∼142, while the 4L sample showed the smallest final GF of ∼22 (all at 3% strain). This large range of values falls both above and below the values of other reported works on similar percolative materials and fabricated devices. For comparison, GF values as high as 448 and 1037 have been reported for 3D filaments and self-assembled thin films, respectively,^54,57^ and other values similar to this work are reported for spray-coated films of graphene flakes,^58^ surfactant-modified graphene flake films,^55^ and a composite sensor fabricated through direct writing of PDMS microbeads combined with electrochemically exfoliated graphene oxide.^59^

To quantitatively compare the different samples, the following metrics were chosen and are tabulated in Table 1: the minimum Δ*R*/⟨*R*_0_⟩ value listed is the most negative change in resistance of the unstrained sample, occurring during the last few cycles. The more negative this number, the more the resistance decrease after straining. The minimum difference between strained and relaxed Δ*R*/⟨*R*_0_⟩ is representative of the smallest strain sensitivity exhibited, with smaller numbers representing less resistance change between relaxed and strained states. Finally, the strain cycle, where 90% of the minimum Δ*R*/⟨*R*_0_⟩ value occurs was recorded to differentiate the initial resistance drop transitioning to a more stable oscillation. At cycles earlier than this value, the sample is at a state of non-equilibrium, with the graphene flake movement induced by the substrate strain. At cycles later than this value, the sample can be considered to be in a state of equilibrium, where the surface particle movement does not significantly change with additional straining cycles.

The contrast between the resistance–strain curves for the samples in Figure 7a–c and the values in Table 1 can be understood as an effect of the amount of platelet material that is on the substrate. During the initial deposition of graphene flakes during dip-coating, the flakes are arranged in a stacked collection on the surface of the PDMS (Figure 7d); being complex geometric shapes, the flakes tend to not stack orderly, resulting in variable packing of flakes that scales with the layer thickness (Figure 7e). During the application of strain, the flakes in immediate contact to the PDMS are displaced relative to their unstrained position due to vdW adhesion between the basal plane of the graphene flakes and the substrate. As the distance between flakes increases (until the maximum substrate strain is reached), the number of conductive pathways is reduced, and the resistance to electron tunneling increases due to the less overlap between particles. This results in the resistance change as reported by a number of previous experimental and theoretical studies.^21,54,58,60,61^ The transfer of shear forces and strain from the substrate to the initial layer of flakes and then to the remaining layers of flake material (next-nearest neighbors and beyond) results in shuffling and reorientation/realignment motions.

SEM of the surface before straining shows a higher occurrence of flakes, which are aligned out-of-plane in different angles and orientations (Figure 8a). Strained samples exhibit a higher degree of packing, which is most apparent for the 2L and 3L samples compared to the 4L sample (Figure 8b). Under in situ strain, cracks are seen on the surface of the samples, which were recoverable upon release of the substrate strain. Under strain, the cracks which open allow space in which the overlaying material can fill into (Figure 8c). The final film structure thus possesses an increased packing density due to the cyclic strain forcing gaps and pockets between flakes to be filled in by subsequent material from the layers above. As the strain is relaxed, the flakes can be understood as being in a more “optimized” position than they were prestrain, resulting in the cycle-induced reduction in resistance.

This behavior of the resistance with cyclic straining is in contrast to other reports, which have utilized for the strain processing of carbon nanotube films deposited on flexible substrates as such works show strain invariable performance after a single strain-conditioning step,^62^ or highlight that the resistance irreversibly increases when strained to a given amount as a result of “strain history” being embedded in the material network.^63^ These differences may be a result of the 2D versus 1D morphology (as in the case of flakes vs nanotubes); more readily though this difference may be rooted in the multilayer dip-coating process applied during the fabrication of samples. For the lowest thickness sample (2L), the sparser concentration of platelet materials allows sufficient free space to maneuver as well as a high potential for open space on the substrate for flakes to settle into, resulting in a significant change in resistance compared to prestraining due to a higher packing density, as shown by the negative Δ*R*/⟨*R*_0_⟩ value in Table 1. The same is seen for the 3L sample, which has a slightly larger resistance change from the 2L. Despite the 3L sample having a greater thickness compared to the 2L sample, it has not reached a “critical thickness”, where the free space mobility of platelets on the surface and in the body of the film is reduced due to the natural tendency for the most efficient packing that would occur if the film thickness continued to increase. The 3L sample begins to show potential for additional reorganization regions from the two spikes in resistance seen around cycles 63 and 75 in Figure 7b. The 4 h 4L sample shows a reduced drop in the maximum resistance change, presumably due to the larger dip-coating layers causing a natural densification and packing of the flakes. As the packing density of the flakes would already be higher than for the 2L and 3L, the maximum degree at which the resistance may be affected by strain decreases. This transition with the 4L samples is also supported by the low GF values compared to 2L and 3L and the increased cycle value when the transition between transient and stable resistance regimes occurs, indicating a more gradual reduction in the film resistance.

Additional examination of the surface roughness of the samples with and without cyclic strain reveals corresponding changes to the samples (Figure 9). When comparing the roughness (Ra) of the samples before and after straining, the application of tensile strain appears to correlate with a reduction in the roughness, particularly for the 2L and 3L samples. The significant decrease in the surface roughness, that is, a smoother sample surface supports the hypothesis of flake packing and reorientation as a result of substrate straining, as well as the densification observed in Figure 8. The 4L sample shows a different trend than the others when strain is applied though, with the surface roughness increasing instead of decreasing after straining. This may be a result of exfoliated flakes being pushed and oriented out of plane due to overcrowding of the film surface, which would correlate with a more gradual reduction in film resistance (higher cycle number, where 90% stability occurs), the smaller minimum difference between strained and unstrained Δ*R*/⟨*R*_0_⟩ values, and the less noticeable SEM contrast after straining.

When comparing the change in surface roughness with and without straining with the extracted resistance strain values in Table 1, a few different correlations can be drawn. First, the amount of resistance reduction (minimum Δ*R*/⟨*R*_0_⟩) appears to positively correlate with the degree of roughness in the final samples. The rougher the strained samples, the lesser the resistance reduction is. For example, the 3L sample exhibits the lowest roughness poststraining and shows the largest decrease in electrical resistance. This correlation can be contributed to higher packing density of flakes. The higher flake packing density after straining gives a smoother surface-improved conduction through a greater contact and an overlap between flakes. Second, the minimum difference between the strained and relaxed Δ*R*/⟨*R*_0_⟩ appears to positively correlate with the initial roughness of the samples. For samples with low initial roughness such as the 4L, this may indicate that many flakes are already well packed together and are less sensitive to strain as opposed to samples with higher initial roughness, where there is a higher sensitivity of the resistance to strain.

## Conclusions

Graphene flakes exfoliated in ethanol and CAB were characterized with respect to their morphology and percolative electrical properties in preparation for application toward the fabrication of conductive coatings on flexible and stretchable PDMS substrates. Resistance–strain measurements combined with extracted resistance-cycle parameters show correlations with sample surface roughness before and after tensile strain cycling. These suggest the role that roughness has with regard to surface reorganization and increased flake packing density that contribute to electrical modification of these types of percolative films.

Overall, the degree of resistance reduction can be enhanced for higher roughness samples below a certain critical thickness, where the platelet particles possess a large amount of free space through which reorientation forces, driven by strain transfer from the substrate, can be allowed to act. Increases to the packing density of the graphene flake films through application of cyclic straining is a low-energy process step with potential for enhancing the performance of films on various stretchable substrates. Conceivably, the application of cyclic straining as a manufacturing routine can be incorporated in wearable and flexible electronic fabrication in several different ways, such as alternating between cyclic straining and film layer deposition or the application of cyclic bending strain for substrates which cannot tolerate as high tensile strain as PDMS. Additional postprocessing methods may also be employed following strain-induced optimization of the deposited flakes in order to “lock-in” their position, such as depositing an overlaying polymer coating. Future work incorporating this technique may also look at the degree of which the conductivity may be improved with the magnitude of the strain applied. The low change in the gauge factor observed for the 4L sample measured also suggests that such processing routes may lead to a reduction in strain sensitivity, which while undesirable for applications related to a variety of mechanical/pressure sensors and detectors may find application as conductive interconnects of wearable devices, where large changes in electrical resistance due to strain would be undesirable.

## Materials and Methods

### Procedure for Exfoliation of Graphene Flakes

Graphite powder (Sigma-Aldrich, 325 mesh) was used as the source material to produce graphene flakes. The powder was used as received and dispersed into a liquid medium consisting of EtOH and CAB. In detail, CAB (10 mg mL^–1^) was dissolved in a closed beaker containing EtOH on a hot plate under magnetic stirring set to 65 °C until a clear solution was obtained. The graphite powder (40 mg mL^–1^) was added and mixed briefly to obtain a homogeneous dark/black mixture. This graphite mixture was transferred in 20 mL aliquots to Falcon centrifuge tubes for the next sonication step. The exfoliation took place in a bath sonicator (Branson 2510, ∼40 W output) for 2- and 4 h intervals to evaluate the influence of the sonication time on the final exfoliation effectiveness as well as the impact on the material’s use in percolative conductive films. Following sonication, the mixtures were centrifuged in three 30 min steps: at 500, 2000, and 4400 rpm using an Eppendorf 5702 centrifuge. After the first two steps, the sediment was collected and set aside, while the supernatant underwent the final centrifugation stage. After the final centrifugation, the sediment was washed three times in pure EtOH at 4400 rpm to remove the excess CAB. The final graphene dispersions were obtained by redispersing the washed flakes in 5 mL of EtOH via brief sonication.

### Modified LB-Dip-Coating Deposition of Flakes on SiO_2_/PDMS Substrates

The deposition process setup was assembled as follows: sample holders (Supporting Information Figure S1) were 3D-printed ad hoc for the coating setup; the substrate was held at a 33.7° angle with respect to the horizontal plane, providing sufficient control over the coating process through a KSV Dip Coater LM.

For graphene flake characterization, 1 × 1 cm^2^ SiO_2_/Si wafers (University Wafers, 300 nm oxide layer thickness) were cleaned by bath sonication in 2-propanol for 5 min. PDMS (SYLGARD 184) substrates were cast in 3D-printed molds and cured following the manufacturer’s instructions and cut into desired dimensions (∼36 × 7 × 1 mm) using a cleaned razor blade. SiO_2_ and PDMS were dip-coated using the same procedure with their respective sample holders. In detail, the procedure steps are as follows: (1) lowering the sample on the holder underneath the water level, (2) addition of the low γ-phase of EtOH/graphene dispersion on the water surface using a micropipet for a total volume of 200 μL, (3) waiting for 1 min to allow the surface turbulence to subside and the flake position to stabilize, and (4) retraction of the sample at 25 mm/min until the entire substrate became coated with the material and free of water. The deposited graphene/EtOH dispersion was left to dry at room temperature for 5–10 min before repeating the procedure to achieve the target number of process layers. Graphene/SiO_2_/Si samples were heated at 500 °C for 60 min in ambient air in a KSL-1100X furnace with an 8 °C/min ramp rate. Graphene/PDMS samples were thermally treated at 175 °C for 60 min with a 6 °C/min ramp rate. Sample cooling to room temperature was performed passively in the closed furnace.

### Characterization of Exfoliated Graphene and Percolative Films

Graphene exfoliated in CAB and graphene thin films were characterized using Raman spectroscopy (Renishaw inVia Raman microscope, 532 nm laser, 50× objective, 1 μm spot size) and XPS (25.7 W, 15 kV, monochromated Al Kα X-rays (1488.6 eV) using a PHI 5700 system with an Omni Focus V lens, a 100 μm spot size, and a 45° takeoff angle). Raman spectroscopy data were collected at 10% maximum power to minimize the thermal effects and possible damage from the laser. Samples for Raman analysis of exfoliated flakes were prepared via filtering exfoliated dispersions of graphene in CAB/EtOH through PVDF filters (0.2 μm pores, 25 mm diameter, Sterlitech), followed by washing in EtOH and redispersion via sonication in fresh EtOH. Washed flake dispersions were then drop-cast onto SiO_2_ wafers for analysis. CVD-grown graphene films were used as a reference Raman sample (Graphenea, monolayer graphene on 300 nm SiO_2_/Si, 1 × 1 in.). For samples prepared through surface-assembled dip-coating on SiO_2_, optical profilometry (Bruker ContourGT KO Optical Profiler, VSI mode, 10× objective), SEM (JEOL JSM 7600F), and four-point electrical analysis (OSSILA Four-Point Probe System, 1.25 mm probe spacing) were conducted in addition to Raman and XPS spectroscopy.

### Electrical and Surface Morphology Measurements for Strain Reorganization

For electrical resistance and strain measurements of graphene flakes on PDMS substrates, an ADMET tensile tester (ADMET MTESTQuattro) was used for applying cyclic tensile straining to the PDMS samples, while two-point resistance measurements were logged using a Hewlett Packard 34401A multimeter and a digital multimeter module in the Keysight BenchVue application. A sawtooth profile was applied for 100 cycles at a rate of 17.5 μm s^–1^, with the initial and final positions set to give a maximum strain of 3% for all samples measured. Copper tape was used for the electrical contacts and was clamped onto the PDMS samples at a separation distance of ∼5.21 mm for all samples. 15 s of resistance data was logged by the multimeter before the strain cycle was initiated to obtain an average resistance for normalizing the resistance change. Optical profilometry data and the SEM images of the samples on PDMS were collected using the same instruments as those used for the samples on SiO_2_/Si wafers..

## Supporting Information Available

Images of the experimental setup; thermal heating data for the CAB polymer; SEM images of precursor graphite; TEM of exfoliated flakes; Raman spectra; XPS spectra; sheet resistance measurements; and optical profilometry data (PDF)
