# Supplementary material for: Graphene Flake Self-Assembly Enhancement via Stretchable Platforms and External Mechanical Stimuli
Source: ACS Omega. 2021 Nov 5;6(45):30607–17. doi: 10.1021/acsomega.1c04368 (PMC8600623; doi:10.1021/acsomega.1c04368)
Supplement: Supplementary file 1 — ao1c04368_si_001.pdf [file ao1c04368_si_001.pdf]

Supporting Information

Supplemental Images and Characterization Data

Graphene Flake Self-assembly Enhancement via Stretchable Platforms and External Mechanical Stimuli

Harrison A. Loh[1], Claudio Marchi[2], Luca Magagnin[2] Konstantinos A. Sierros[1]*

[1]West Virginia University, Morgantown, WV, USA

[2]Department of Chemistry, Materials and Chemical Engineering Giulio Natta, Politecnico di Milano, Via Mancinelli 7, 20131 Milano, Italy

Supporting Info Section 1: 3D Printed Sample Holder for use with Dip-coater

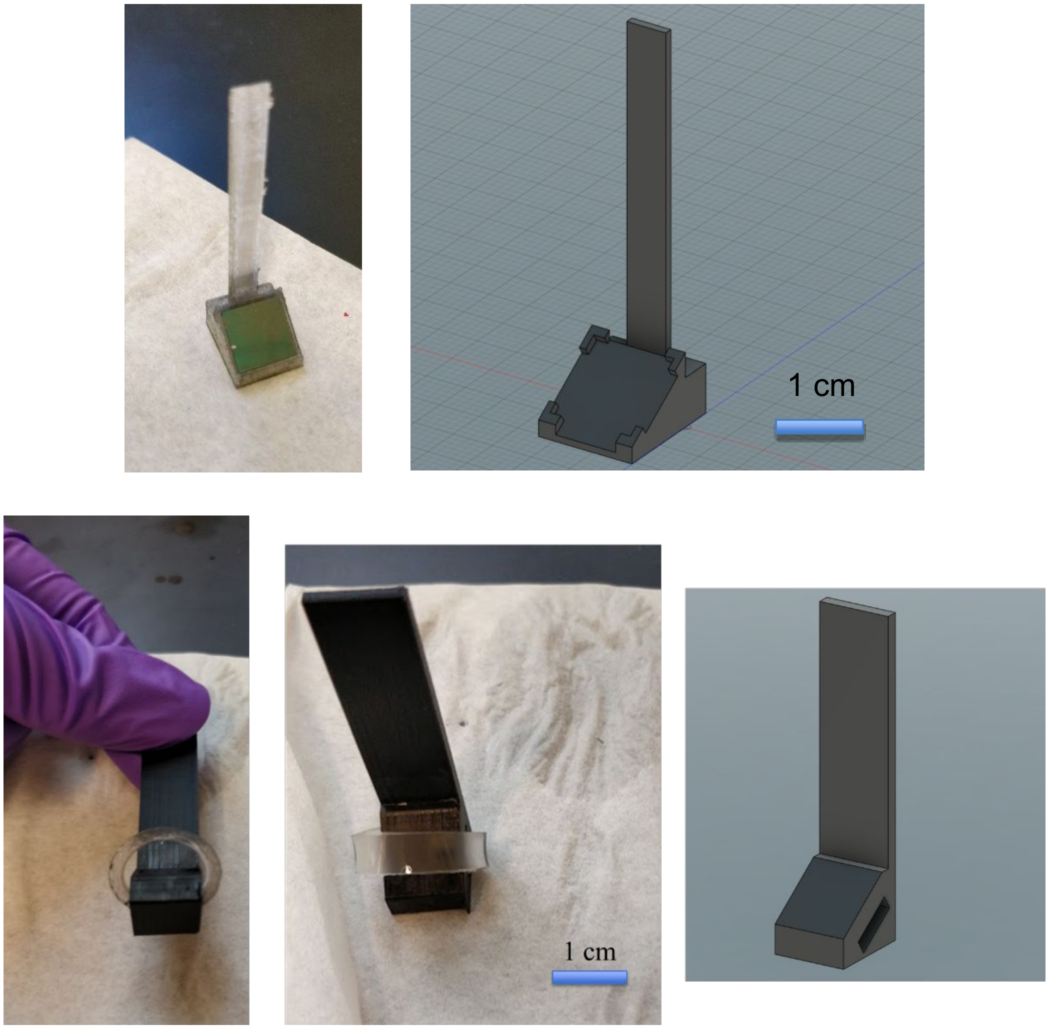

Figure S1. 3D printed sample holders employed for dip coating. (Top) Holder for SiO_2 substrates and (Bottom) for flexible PDMS substrates (the images here were all taken by the authors).

3D printed sample holder was printed using commercial PLA filament using an Ultimaker 2+ printer. Autodesk Fusion 360 was used to design the CAB and generate the STL file for printing with Cura for the slicing into gcode.

Supporting Information Section 2: TGA of Cellulose Acetate Butyrate (CAB)

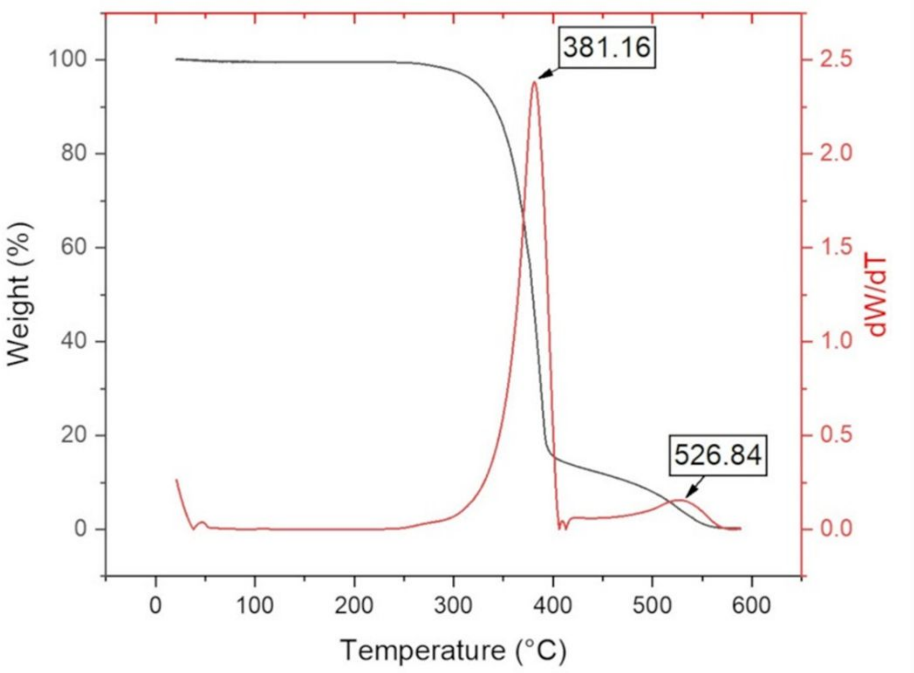

Figure S2. Thermogravimetric Analysis of plain CAB polymer. Initial decomposition is shown to occur at approximately 380°C.

Supporting Info Section 3: SEM of precursor graphite powder for exfoliation

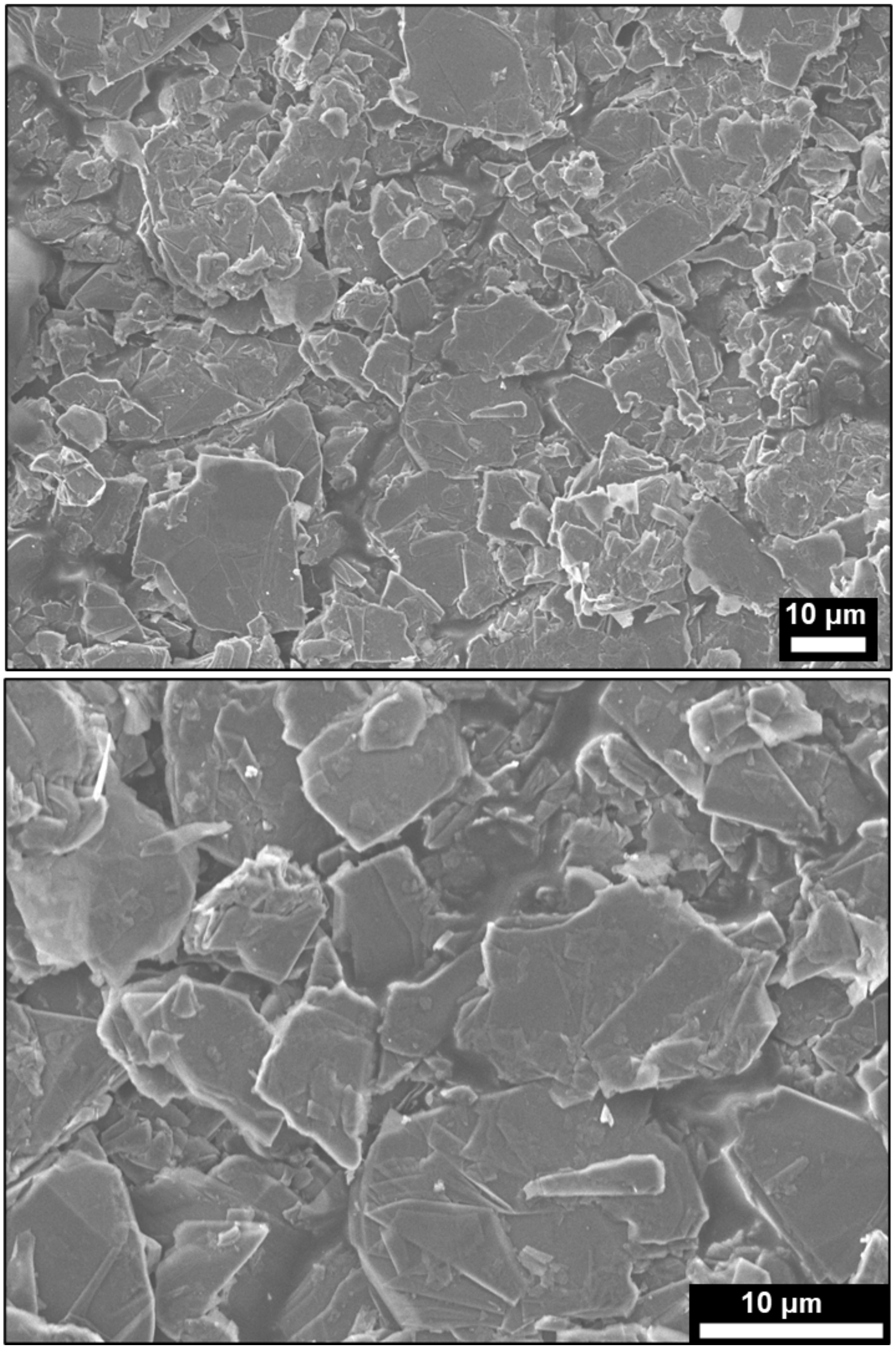

Figure S3. SEM of initial 325-mesh graphite powder prior to exfoliation in CAB/EtOH. Large flake sizes with respect to the Raman spectroscopy laser result in a small intensity of the D peak from low flake edge contributions.

Supporting Info Section 4: Raman spectroscopy of deposited film samples

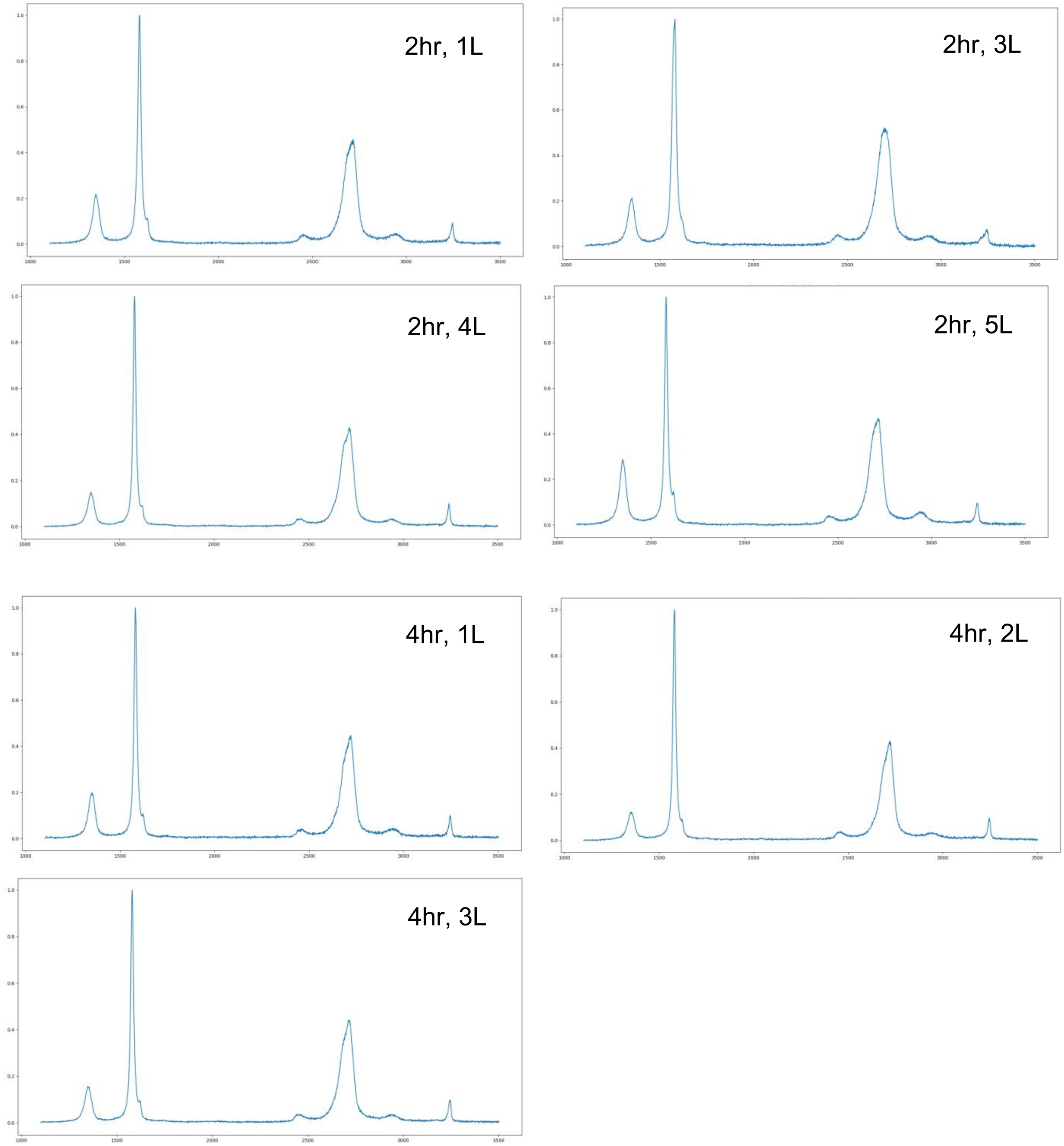

Figure S4. Representative Raman Spectra from film samples using different hour sonicated material and with different dip-coated layers of material deposited.

Supporting Information Section 5: XPS Survey Spectra of Self-assembly deposited films

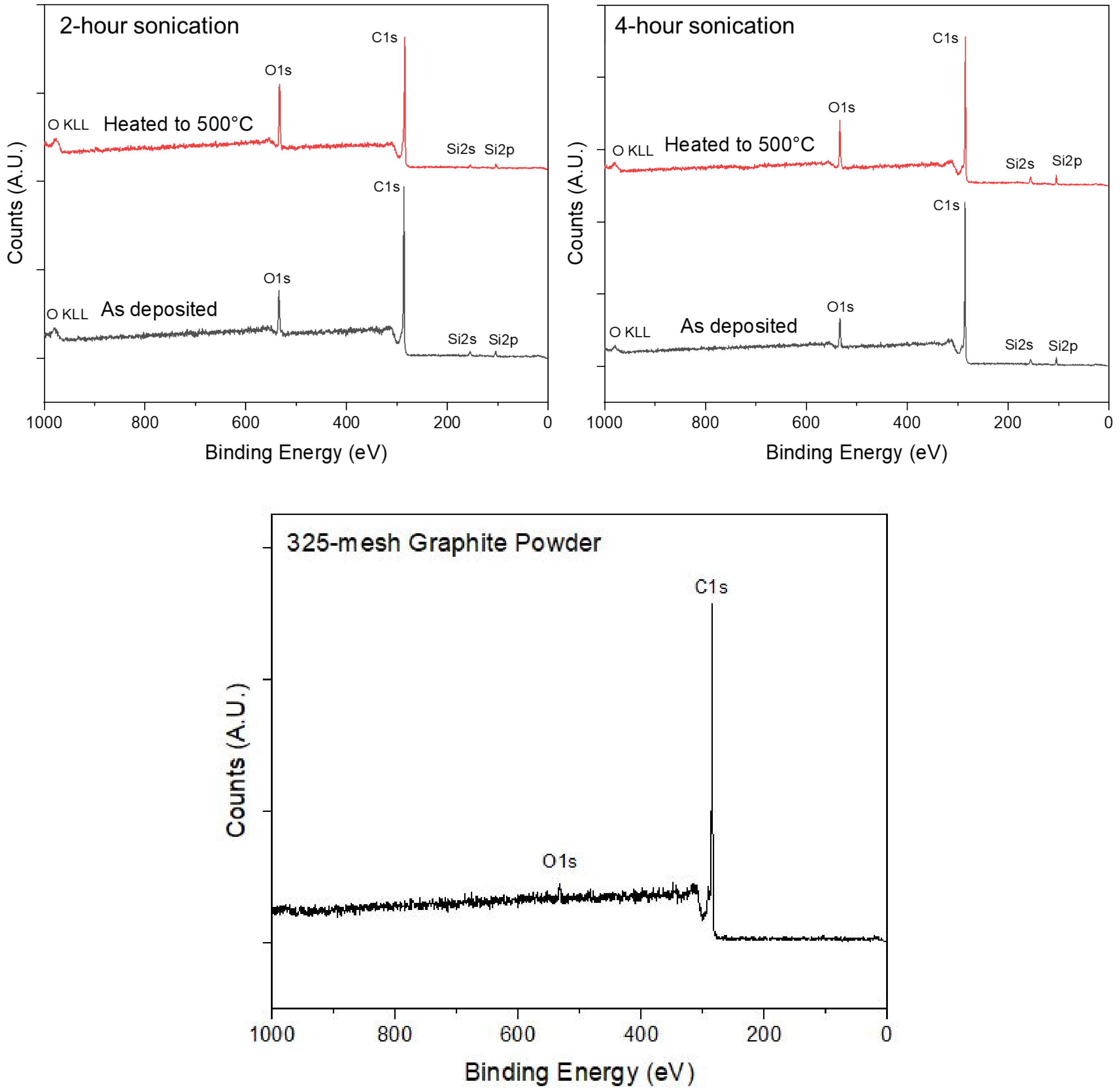

Figure S5. Survey XPS spectra collected from a 2hr and 4hr exfoliated film sample. Peaks shown are consistent with carbon flakes deposited onto a SiO_2/Si wafer. XPS Survey of precursor graphite powder shows a minimal O1s peak, suggesting oxidation originating from either the substrate or the exfoliation process.

Supporting Information Section 6: High-res C1s XPS of 2hr exfoliation and 4hr exfoliated material

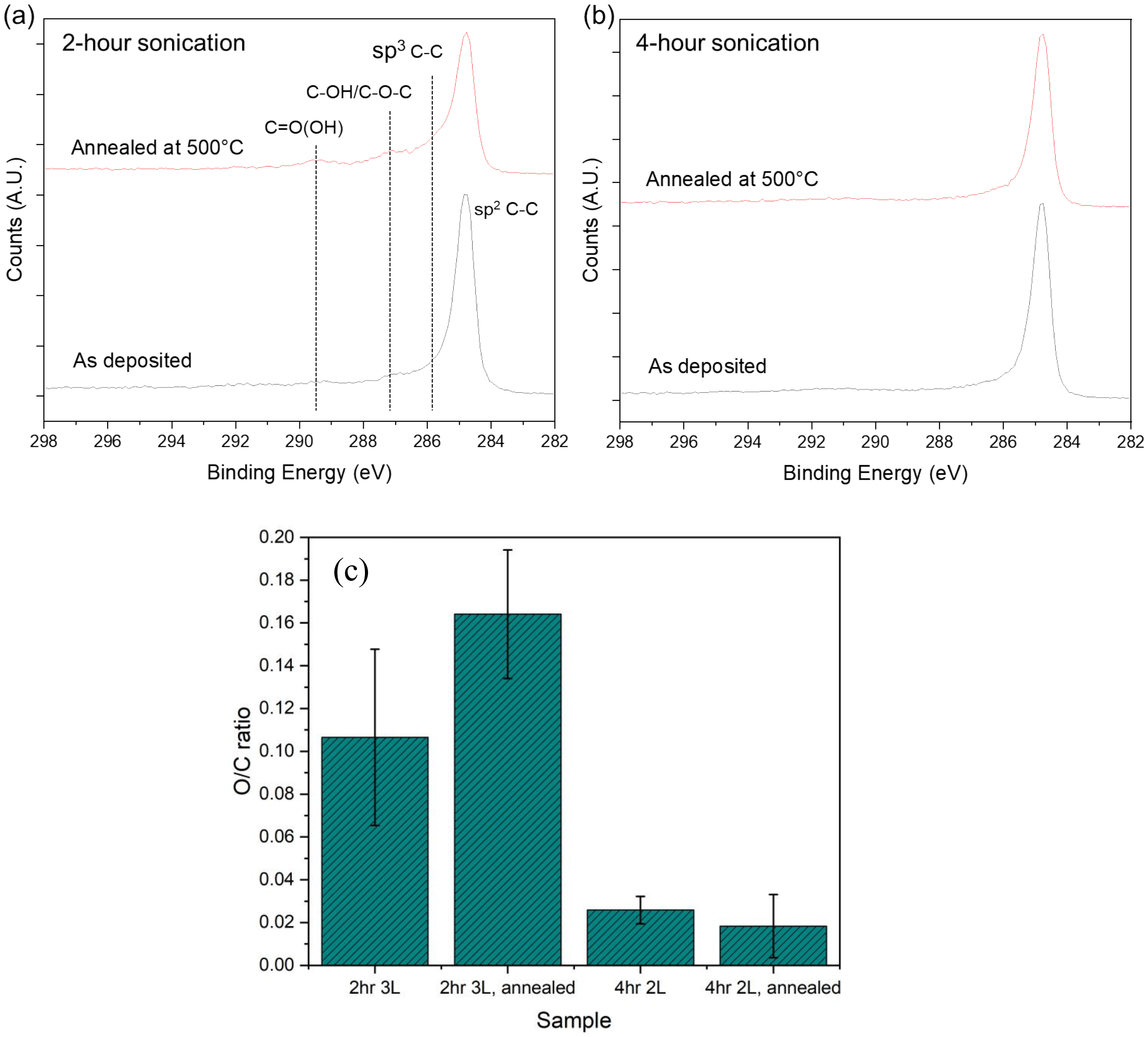

Figure S6. (a, b) High-resolution spectra of 2hr and 4hr exfoliated films before and after heating to 500°C in ambient air for 1hr. Additional peaks that arise in the 2hr sample are assigned to carboxyl groups (C=O(OH)), hydroxyl/epoxide groups (C-OH and C-O-C), and formation of addition sp3 C-C bonds in the graphene basal plane. (c) O/C ratios for 2hr sonicated samples compared to 4hr sonicated samples were also calculated for comparison. The ratio of O/Si was determined from measurements of an SiO_2 wafer cleaned via sonication in EtOH followed by DI water and air drying. This oxygen contributions from the underlying SiO2 substrate were subtracted from the as-measured elemental content.

Supporting Information Section 7: Sheet Resistance of Graphene Flakes Films on SiO_2/Si

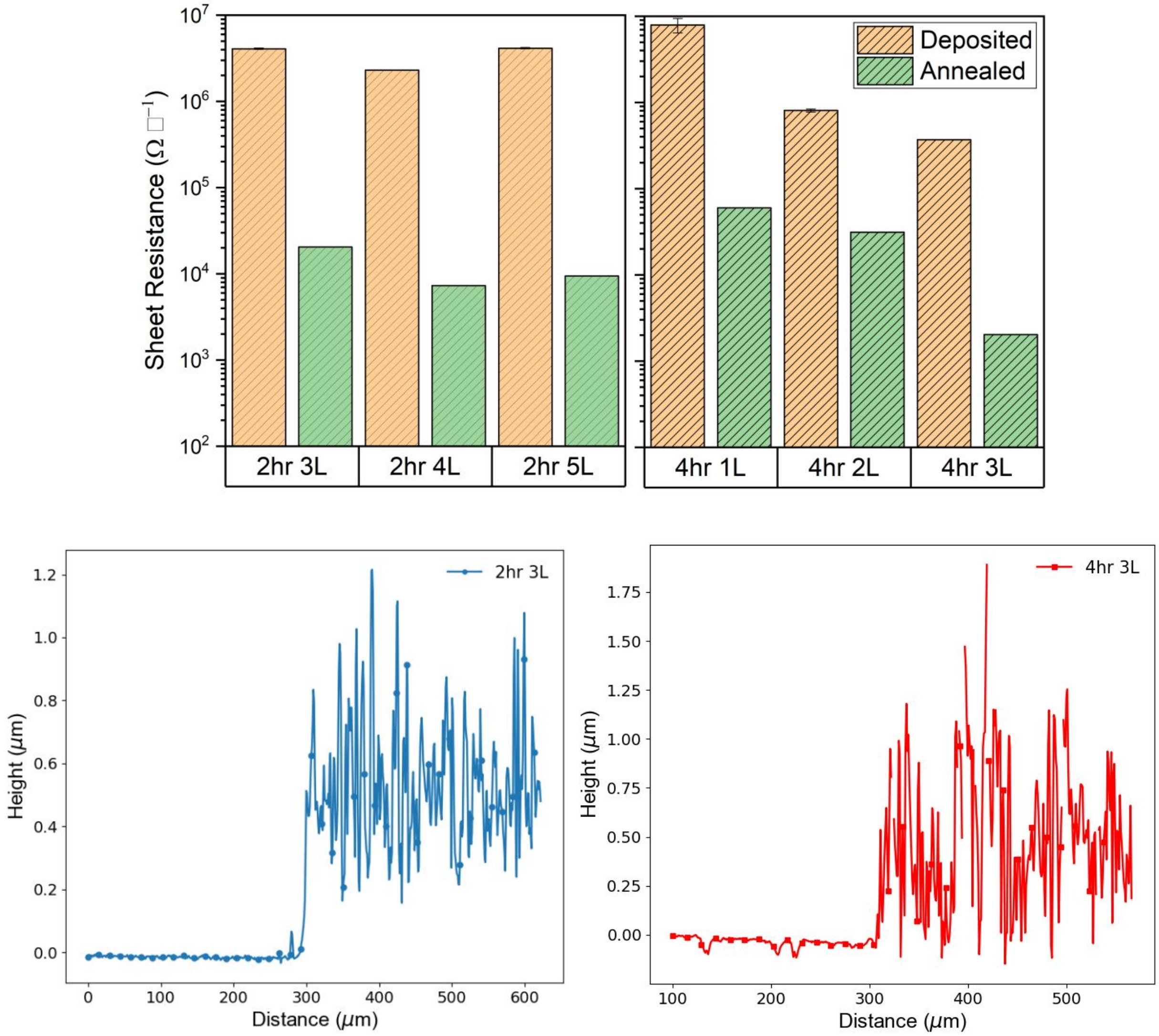

Figure S7. (Top) 4-pt. sheet resistance measurements for 2hr and 4hr sonicated graphene flake percolative films on SiO_2/Si for different number of dip-coating iterations. The 4 hour sonicated flakes show a more consistent reduction of resistance with an increase in the film thickness, both as deposited as well as after annealing at 500°C. (Bottom) Representative profilometry profiles of 2hr and 4hr exfoliated flakes at edge step areas of the deposited films on SiO_2/Si. Average steps heights were determined in order to convert sheet resistance values into conductivity measurements.

Supporting Information Section 8: Optical Profilometry Map of Pre and Post Strained Graphene films on PDMS

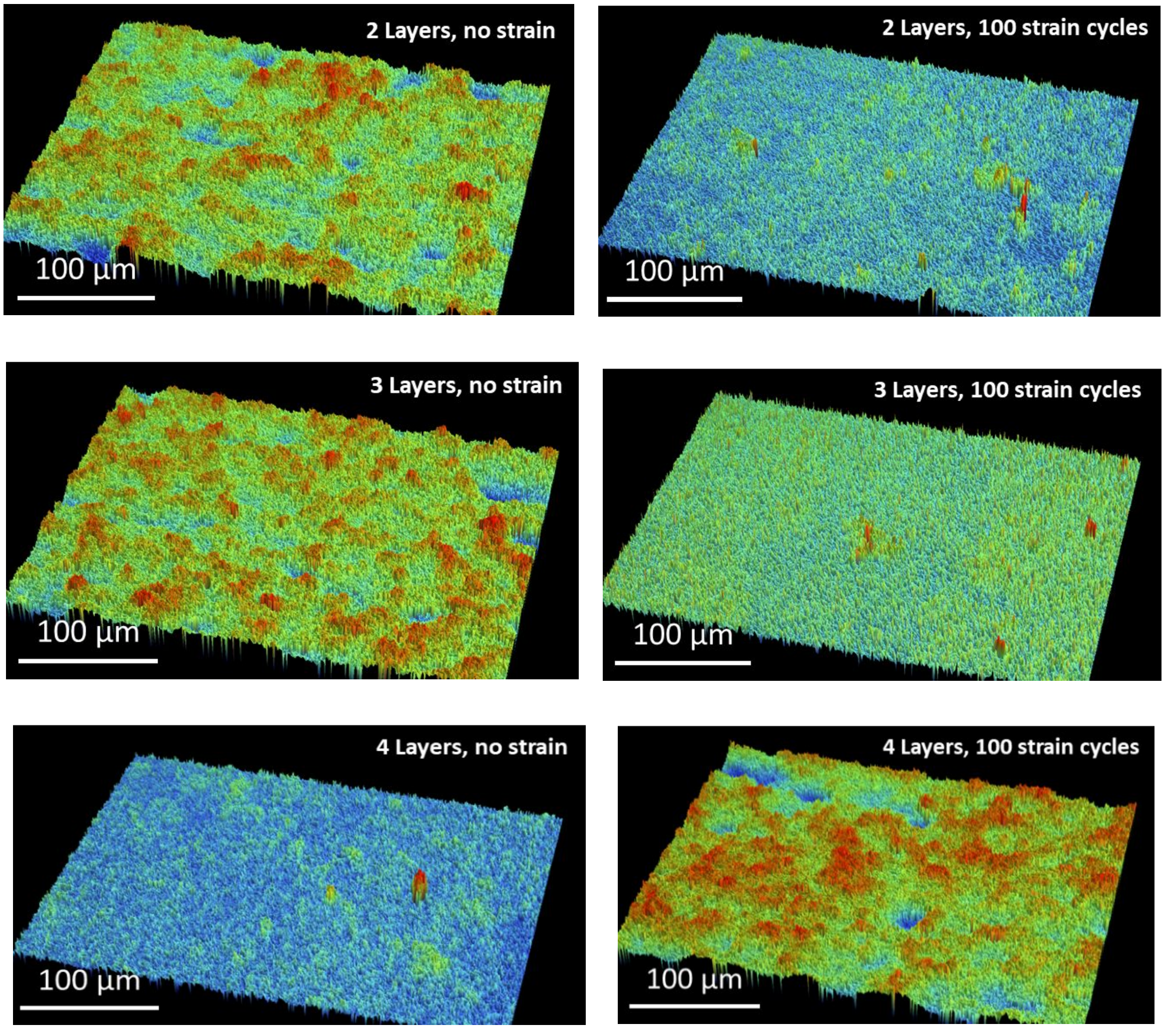

Figure S8. Optical profilometry maps from which surface roughness values of samples before and after straining on PDMS were extracted. Like the corresponding SEM images, the surface roughness of 2- and 3-layer samples became smoother following 100 cycles of tensile strain, whereas the 4-layer sample increased in roughness after 100 cycles of strain.
